# Therian origin of INSL3/RXFP2-driven testicular descent in mammals

**DOI:** 10.3389/fcell.2024.1353598

**Published:** 2024-02-02

**Authors:** Brandon R. Menzies, Gerard A. Tarulli, Stephen R. Frankenberg, Andrew J. Pask

**Affiliations:** School of BioSciences, Faculty of Science, The University of Melbourne, Melbourne, VIC, Australia

**Keywords:** testicular descent, scrotum, marsupial, dunnart, development

## Abstract

**Introduction:** During early development in most male mammals the testes move from a position near the kidneys through the abdomen to eventually reside in the scrotum. The transabdominal phase of this migration is driven by insulin-like peptide 3 (INSL3) which stimulates growth of the gubernaculum, a key ligament connecting the testes with the abdominal wall. While all marsupials, except the marsupial mole (*Notoryctes typhlops*), have a scrotum and fully descended testes, it is unclear if *INSL3* drives this process in marsupials especially given that marsupials have a different mechanism of scrotum determination and position relative to the phallus compared to eutherian mammals.

**Methods:** To understand if INSL3 plays a role in marsupial testicular descent we have sequenced and curated the *INSL3* gene and its receptor (*RXFP2*) in a range of marsupials representing every order. Furthermore, we looked at single cell RNA-seq and qPCR analysis of INSL3 in the fat-tailed dunnart testis (*Sminthopsis crassicaudata*) to understand the location and timing of expression during development.

**Results:** These data show a strong phylogenetic similarity between marsupial and eutherian orthologues, but not with monotreme INSL3s which were more similar to the ancestral RLN3 gene. We have also shown the genomic location of *INSL3*, and surrounding genes is conserved in a range of marsupials and eutherians. Single cell RNA-seq and qPCR data show that *INSL3* mRNA is expressed specifically in Leydig cells and expressed at higher levels during the testicular descent phase in developing marsupials.

**Discussion:** Together, these data argue strongly for a therian origin of INSL3 mediated testicular descent in mammals and suggests that a coordinated movement of the testes to the abdominal wall may have preceded externalization in marsupials and therian mammals.

## Introduction

Testicular descent is present in more than 99% of extant mammals and is thought to have arisen due to the evolution of endothermy and the resulting temperature-induced negative effects on sperm production (Werdelin and Nilsonne, 1999; Kleisner et al., 2010). However, there are numerous eutherian mammals, namely within Afrotheria, with either partial testicular descent or internal testes (testicond), while both living species of monotremes are testicond (Sharma et al., 2018). Thus, the evolution of testicular descent and externalization of the testes in mammals is complex and difficult to reconstruct phylogenetically.

Testicular descent occurs in two phases: a transabdominal phase where the testes migrate from a position near the kidneys to the lower abdomen, and an inguinal phase where they move through the abdomen via the inguinal canal into the scrotum (Foresta et al., 2008; Hutson et al., 2015). Insulin-like peptide-3 (INSL3) is expressed by Leydig cells of the testis and is crucial for the transabdominal phase of testicular descent, and loss of function causes the testes to remain in the ovarian position near the kidneys in mice (Zimmermann et al., 1999). Furthermore, the overexpression of INSL3 in female mice results in sub-abdominal ovaries (Adham et al., 2002). A number of afrotherian mammals that have lost testicular descent have premature stop or nonsense mutations in either *INSL3* or its receptor *RXFP2*, making them non-functional (Sharma et al., 2018), demonstrating a secondary loss of this pathway as opposed to its absence in these species.

INSL3 is most closely related to the relaxin-3 peptide in mammals and evolved via the duplication of a specific relaxin-like family locus (RFLC-I) that occurred in the last common ancestor of teleosts and tetrapods approximately 450 million years ago (Park et al., 2008). RFLC-I was the ancestral locus that became the *RLN3* gene in mammals. Its duplication created RLFC-II, which became *INSL3* in therian mammals. The role of INSL3 in testicular descent appears to have evolved in the ancestor of therian mammals not only because monotremes have internal testes and lack a gubernaculum but also because monotreme *INSL3* does not group with therian *INSL3* sequences phylogenetically and is more similar to mammalian *RLN3* sequences (Park et al. 2008). Furthermore, a specific amino acid substitution from arginine (R) to histidine (H) at residue 12 of the INSL3 beta chain is crucial for RXFP2-specific binding and is absent in monotremes (Park et al., 2008).

While all marsupials appear to have some form of testicular descent, the role of INSL3 in this pathway has not yet been evaluated other than to show that *INSL3* is present in the genome of the gray short-tailed opossum (Park et al., 2008). Furthermore, marsupials have a different genetic mechanism for scrotum specification than that of eutherian mammals, whereby its development is genetically regulated and based on X-chromosome dosage and not hormonally dependent as in eutherians (Sharman et al., 1970; Watson et al., 1997). As a result, intersex XXY marsupials have a pouch, phallus, and internal testes, while XO marsupials have a scrotum, ovaries, and no pouch. Furthermore, treatment of neonatal marsupials with either androgens or estrogens does not affect the development of either the mammary glands, pouch, or scrotum (Shaw et al., 1988). Finally, the location of the scrotum in all marsupials is cranial to the penis as opposed to caudal as in all eutherians (Sharman, 1970). Given that the marsupial scrotum may be analogous to that of eutherians but not homologous, it is entirely possible that marsupials may have a different mechanism of testicular descent.

Here, we curated the *INSL3* and *RXFP2* sequences from representatives of every order of marsupials and show that marsupial *INSL3* sequences group phylogenetically with eutherians but not monotremes, while all marsupials have the crucial amino acid substitution that allows RXFP2-specific binding (Arg to His at position 12 of the *INSL3* beta chain). We demonstrated genomic synteny in the *INSL3* position within the genomes of five marsupials. We also showed the Leydig cell-specific expression of *INSL3* mRNA in the fat-tailed dunnart (*Sminthopsis crassicaudata*) and a significant upregulation of *INSL3* mRNA expression during the key period of testicular descent in this species [D30–60 post-partum (pp)]. Combined, these data clearly demonstrate a similar role for INSL3 in marsupial testicular descent and show conclusively that this process evolved in the ancestor of therian mammals at least 160 million years ago.

## Methods

### Collection of pouch young/tissues

All animal procedures, including laboratory breeding, were conducted in accordance with the current Australian Code for the Care and Use of Animals for Scientific Purposes and were approved by the University of Melbourne Institutional Animal Ethics Committee (#10206) and with the appropriate wildlife permits from the Department of Environment, Land, Water, and Planning. Animals were housed in a breeding colony at the University of Melbourne School of BioSciences. Pouch young (PY) were aged from the approximate day (D) of birth and were removed by exposing the mother’s pouch and applying gentle traction between the nipple and mouth while supporting/cradling the head/body using fingertips. PY were placed on a Petri dish on ice to provide ice anesthesia and then decapitated using a razor blade. Gonads were immediately dissected from the PY, snap-frozen in liquid nitrogen, and stored at −80°C for later analysis. These tissues were collected opportunistically from PY killed for other ethically approved experiments. The sex of PY and gonads was determined at the earliest ages (D2 pp and D5 pp) by SRY PCR of genomic DNA and at later ages by visualization of the developing pouch or scrotum.

### Histological analyses

Whole dunnart PY were fixed in 4% paraformaldehyde for 24 h and then washed three times in 1x phosphate-buffered saline for 30 min each wash and then stored at 4°C until processing into wax blocks for sectioning. Sections were taken at 10 μm intervals, mounted on glass slides, and stained with hematoxylin and eosin.

### RNA extraction, cDNA synthesis, primers, and qPCR methods

Total RNA was extracted from frozen gonads using the Sigma GenElute Total Mammalian RNA Tissue Kit (#RTN70-1KT). RNA was then converted to cDNA using the Superscript IV system (Thermo Fisher Scientific, Massachusetts) and OligoDT primers. Primer sequences for this study included dunnart *INSL3* forward (TCT​GTG​CCC​ACA​ACT​TCA​TC) and reverse (AGG​CCA​GCA​GGT​CTT​GTT​T), *RXFP2* forward (AAC​ATT​CGT​CCA​GGA​AAA​CG) and reverse (TGT​TCT​CTG​ATG​CCA​TCT​GC), TATA-box binding protein forward (TBP; TTT​CCC​ATA​AGA​TTA​GAA​GGG​CTG​G) and reverse (GCT​TCA​TAG​ATC​TCT​GCT​CTG​ACC), and pumilio RNA binding family member 1 forward (PUM1; ATG​GAG​CAT​GTT​GGC​ATG​GA) and reverse (CCT​ATG​TGA​GGC​TGA​TGG​GC) and were designed to span introns to eliminate amplification of gDNA. We chose these genes as they have previously been used to compare gene expression levels in the testis. Quantification was performed using a QuantStudio 5 Real-Time PCR System (Applied Biosystems, Massachusetts) and PowerTrack SYBR Green Master Mix (Thermo Fisher Scientific, Massachusetts) in a 10 μL reaction containing 5 μL SYBR, 3 uL sterile water, 0.5 μL each of 10 μL forward and reverse primers, respectively, and 1 μL of diluted cDNA (1:5 in sterile water). Quantitative PCR was conducted with an initial step of 95°C for 20 s, 40 cycles of 95°C for 1 s, and then 56°C for 20 s. Results were calculated using the delta CT method, and the expression was shown relative to the dunnart *PUM1* housekeeping gene*.* We also compared the expression relative to the dunnart TBP housekeeping gene, which showed the same general pattern (Supplementary Figure S1). Data are presented as the mean +/- the standard error, with significant differences determined by the two-sample *t*-test.

### RNA-seq data

Dunnart single-cell RNA-seq data were derived from a larger study to be published in another manuscript. Dissociated single cells from juvenile and adult male dunnarts (*n* = 2 total, pooled) were processed using the Clontech SMART-Seq v4 3’ DE kit. Libraries were prepared using the Illumina Nextera XT DNA Sample Preparation Kit and sequenced at 1 million reads per cell (12 cells per multiplexed library) on an Illumina Nextseq 500 platform. Using the Galaxy web-based analysis platform (https://usegalaxy.org/), de-multiplexed reads were mapped to dunnart genomic scaffolds (S.R.F. and A.J.P., unpublished) using Bowtie for Illumina (default parameters) and assembled into transcripts using StringTie and StringTie merge. StringTie was then reapplied to the mapped reads using the StringTie merge output as a transcript reference file to produce the TPM (transcripts per million) dataset reported here. *INSL3* was identified among the StringTie merge-assembled transcripts using BLASTn. Presumptive somatic and germ cells were identified using the following established markers: Leydig cells (10 in total) were identified by the expression of *steroidogenic acute regulatory protein* (*STAR*), *luteinizing hormone receptor* (*LHR*), and *nuclear receptor subfamily 2 group F member 2* (*NR2F2*), Sertoli cells (8 in total) by *SRY-box transcription factor-9* (*SOX9*), and *claudin-11* (*CLDN11*), spermatocytes (16 cells in total) by *synaptonemal complex protein-1* (*SYCP1*) and *piwi-like RNA-mediated gene silencing-2* (*PIWIL2*), and spermatogonia (14 cells in total) by two or more of the following *POU class 5 homeobox-1* (*POU5F1*), *inhibitor of DNA binding-4* (*ID4*), *NANOG*, *NANOS3*, *GDNF family receptor alpha-1* (*GFRA1*), and *CXC motif chemokine receptor-4* (*CXCR4*). The data are presented as an average of total *INSL3* transcripts per cell between presumptive cell types.

### Sequence curation and phylogenetic analysis

*INSL3*, *RLN3*, and *RLN1* nucleotide sequences for human (*Homo sapiens*), mouse (*Mus musculus*), rabbit (*Oryctolagus cuniculus*), dog (*Canis lupus familiaris*), wombat (*Vombatus ursinis*), koala (*Phascolarctus cinereus*), opossum (*Monodelphis domestica*), Tasmanian devil (*Sarcophilus harrisii*), Monito del monte (*Dromiciops gliroides*), brushtail possum (*Trichosurus vulpecula*), agile gracile opossum (*Gracilanus agilis*), platypus (*Ornythorynchus anatinus*), and echidna (*Tachyglossus aculeatus*) were curated by BLAST search of publicly accessible genomes using NCBI (Table 1). *INSL3* nucleotide sequences for *S. crassicaudata* (GenBank: OR839190) and *Notoryctes typhlops* (GenBank: OR839192) and *RXFP2* nucleotide sequences for *S. crassicaudata* (GenBank: OR839191) were curated by searching unpublished genomes in our laboratory. These sequences have been submitted to GenBank. Sequences were aligned using MUSCLE, and phylogeny was determined using the neighbor-joining method with 1,000 bootstrap replicate analyses using human *IGF1* as an outgroup (MacVector).

**TABLE 1:** Species and associated accession numbers for sequences used in phylogenetic analyses.

*Species*	*RLN1*	*RLN3*	*INSL3*	*RXFP2*
Human	NM_006911.4	AF_447451.1	NM_001265587.2	NM_130806.3
Mouse	NM_011272.2	NM_173184.1	NM_013564.7	NM_080468.2
Dog	NM_001003132.1	XM_038567067.1	NM_001002962.1	NM_001005870.1
Rabbit	NM_001082320.1	NM_001122941.1	NM_001122941.1	XM_017350149.2
Wombat	N/A	XM_027851320.1	XM_043980545.1	XM_027877598.1
Koala	XM_021006313.1	XM_020995055.1	XM_021009096.1	XM_020997197.1
Tasmanian devil	XM_02349710.2	XM_031947695.1	XM_003762760.4	XM_003764504.3
Opossum	XM_007499309.3	XM_001377431.4	XM_007489659.3	XM_007495281.3
Monito del monte	XM_043974591.1	XM_043995161.1	XM_043980545.1	XM_043997436.1
Agile gracile opossum	N/A	XM_044658451.1	XM_044658657.1	XM_044668960.1
Brushtail possum	XM_036738887.1	XM_036752124.1	XM_036751821.1	XM_036744005.1
Platypus	XM_007655587.2	NM_001122689.1	NM_001122688.1	XM_039915010.1
Echidna	XM_038769380.1	XM_038771772.1	XM_038771846.1	XM_038762176.1

## Results

### Histological and macroscopic description of testicular descent and the development of the pouch and scrotum in the fat-tailed dunnart

**FIGURE 1:**
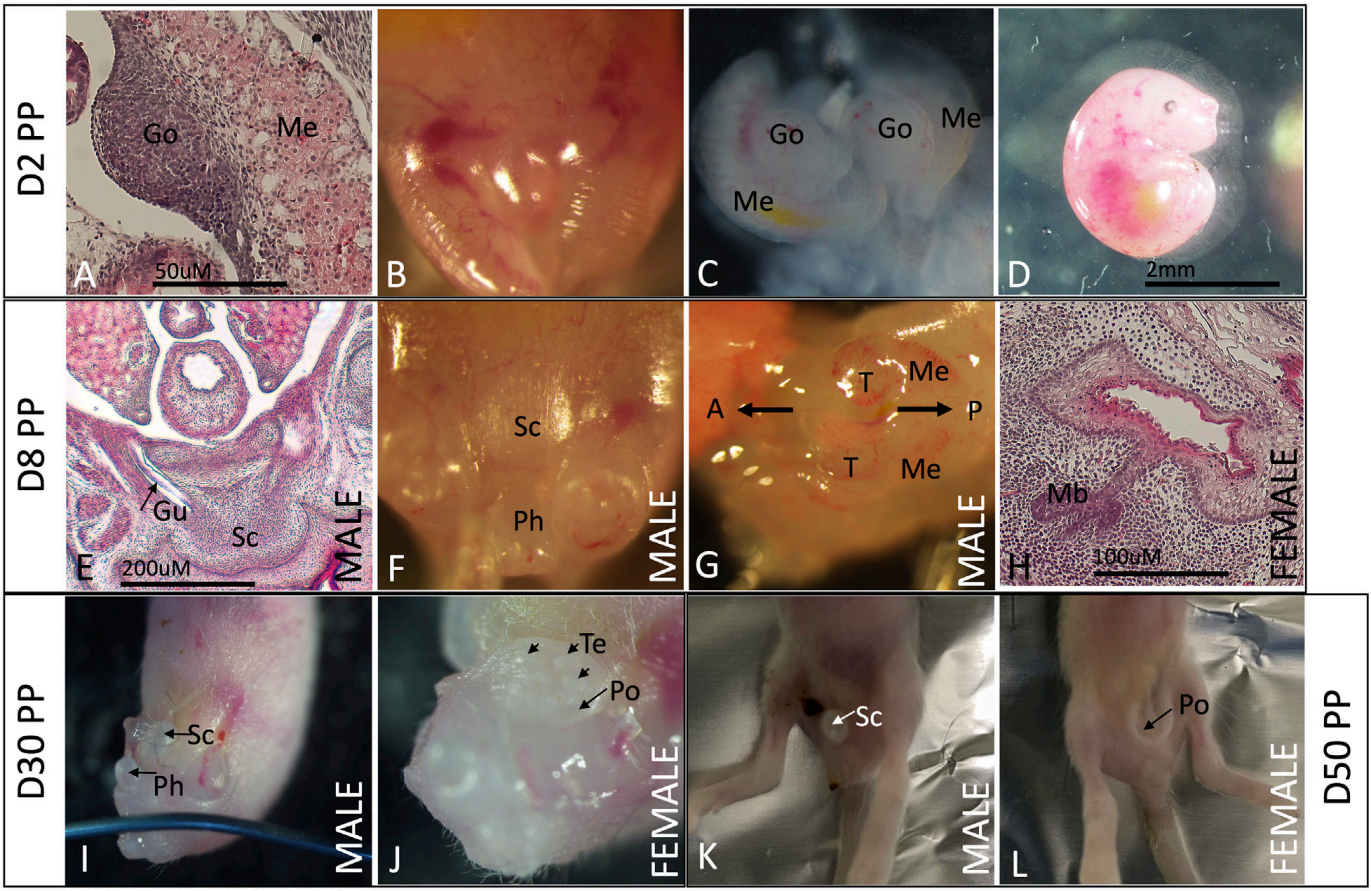
Testicular descent and scrotum/mammary region development in the fat-tailed dunnart. From D2–8 PP, the gonads are intra-abdominal, while the developing scrotal bulge is visible in males by D8 **(A, C, D, E–G)**. At D2, there is very little differentiation of the epidermis that will later form the scrotum in males or the mammary glands and pouch in females **(B)**. In D8 females, the mammary primordia are well differentiated, and mammary buds have started to form **(H)**. By D30, the testes are descending transabdominally, and the scrotum is fully formed **(I)**. Females have visible teats and a pouch lip by D30 PP **(J)**. By D50 PP the testes are inguinal, and the scrotum/pouch is are clearly visible **(K, L)**. Testes fully descend into the scrotum by D60 PP. A: anterior, P: posterior, Go: gonad, Gu: gubernaculum, Me: mesonephros, Mb: mammary buds, Ph: phallus, Po: pouch, T: testis, and Te: teat.

Some details regarding testicular descent in the fat-tailed dunnart have been documented previously from micro-CT data (Cook et al., 2021), including that the mammary primordia differentiate at about D2 PP while the scrotum starts to differentiate at about D4 PP and that the testes are fully descended into the scrotum by D60 PP. Here, we added to that developmental profile with post-natal pictures and descriptions of the gonads and external development of the pouch and scrotum in the dunnart. At D2, the gonads (Go) can be seen as small translucent spheres that sit in an anterior-medial position in the abdomen next to the mesonephros (Figures 1A, C; Me). There is no overt pouch (Po) or scrotum (Sc) structure at this stage (Figures 1B, D). At D8 PP, there is a well-defined scrotal bulge both histologically (Figure 1E) and macroscopically (Figure 1F), and the gubernaculum (Gu) can clearly be seen linking the scrotum with the testes. Up until D8, the gonads and mesonephros increase slightly in size but do not change their position at all within the body cavity (Figure 1G). At D8, the mammary buds are well-developed histologically, but the pouch area is not yet visible on the surface of the abdomen except for some minute indentations of the developing teats (Figure 1H). At D30, the testes are located much lower in the abdomen, separate from the mesonephros but not yet inguinal, while the scrotum is fully formed (Figure 1I). The D30 PP female has a well-defined pouch ring and teats (Figure 1J). By D50, the scrotum and pouch region are very well differentiated (Figures 1K, L).

### Nucleotide sequence and genomic comparisons of marsupial INSL3 and RXFP2

**FIGURE 2:**
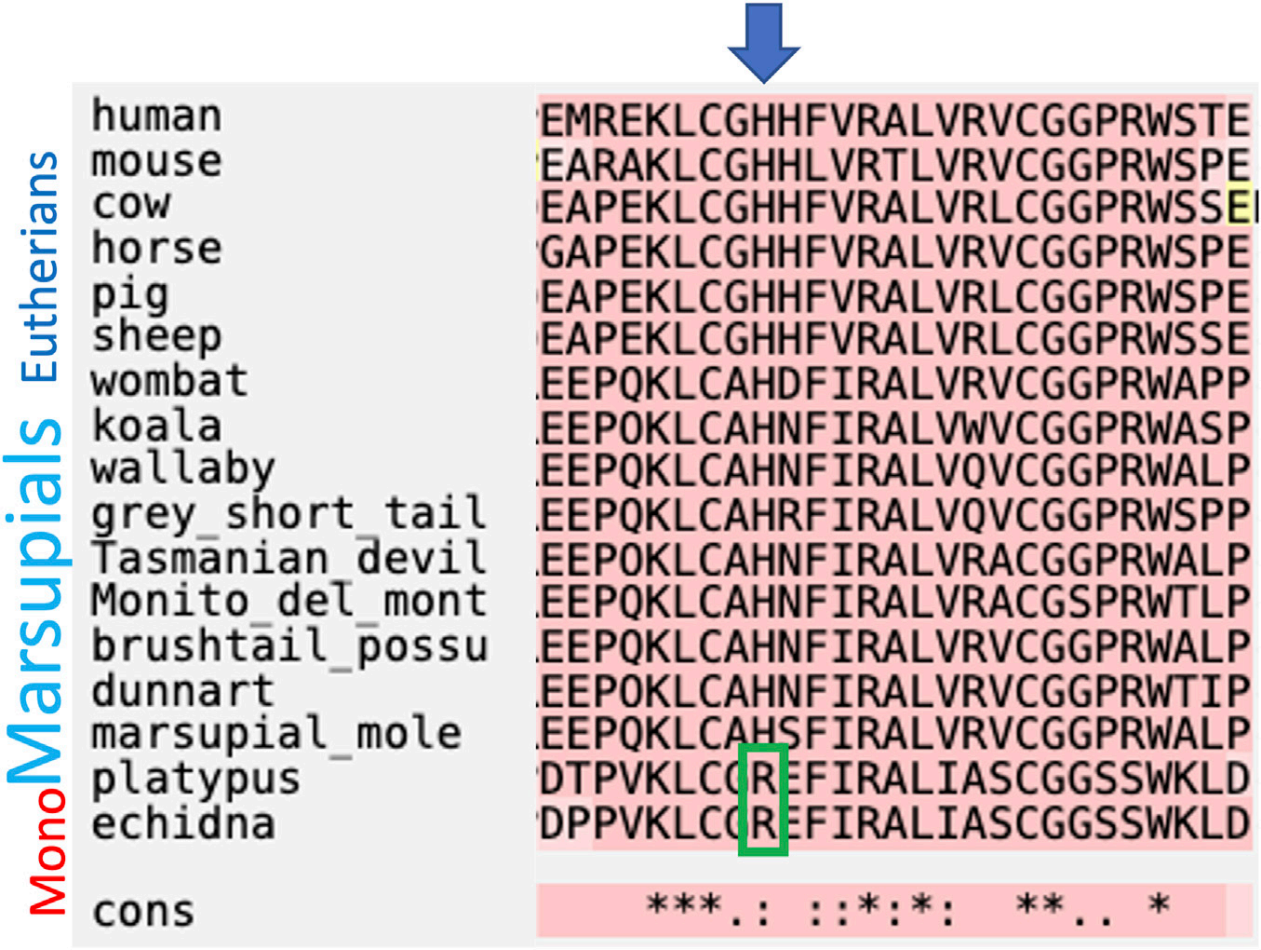
Comparison of amino acid sequences for the INSL3 beta chain between mammals. INSL3 binding to its receptor (RXFP2) is conferred by a single R to H amino acid substitution that is absent present in monotremes.

The full-length coding *INSL3* cDNA and predicted amino acid sequences were not highly conserved among therian mammals overall (approximately 40% and 45% identity, respectively). However, the nucleotide and amino acid sequences for the *INSL3* beta chain, which confers specific binding to RXFP2, were 75% and 80% identical, respectively. Importantly, a key amino acid residue substitution from arginine (R) to histidine (H) at position 12 of the INSL3 beta chain, was demonstrated to confer specificity to RXFP2 (as opposed to both RXFP1 and -2 in monotremes and non-mammalian vertebrates), was conserved in all marsupials relative to eutherians but absent in monotremes (Figure 2).

**FIGURE 3:**
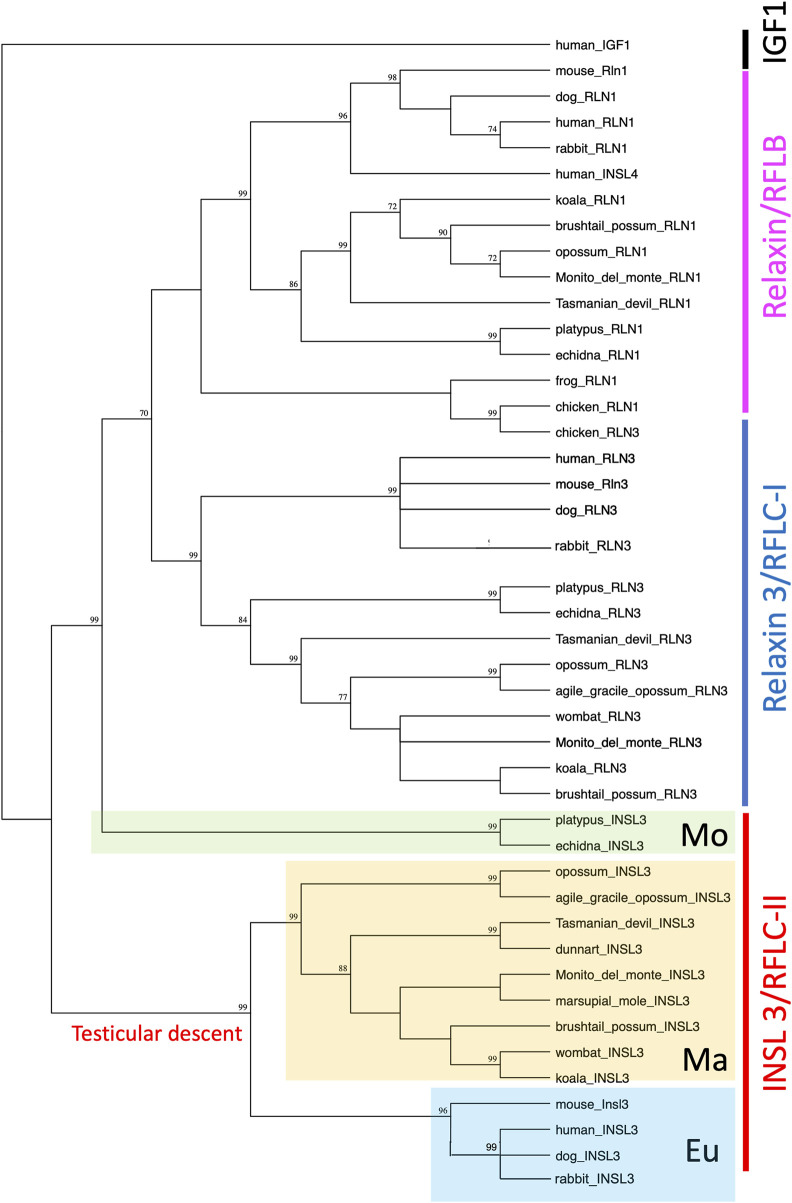
Phylogenetic comparison of the RLN1/RLN3/INSL3 family of sequences in vertebrates. Phylogenetic analysis shows that all therian *INSL3* sequences group together, while monotreme *INSL3s* group separately.

Phylogenetically, all therian *INSL3* (*RFLC-II*) sequences, apart from monotremes, grouped together with strong bootstrap support (99; Figure 3). Monotreme INSL3s then branched off separately to the *relaxin* (*RLN1*/*RFLB*) and *relaxin*-*3* (*RLN3*/*RFLC-I*) sequences, also with strong bootstrap support (99; Figure 3). All mammalian RLN1 and RLN3 sequences were grouped separately, except for chicken *RLN3,* which was grouped with the other non-mammalian *RLN1* sequences.

**FIGURE 4:**
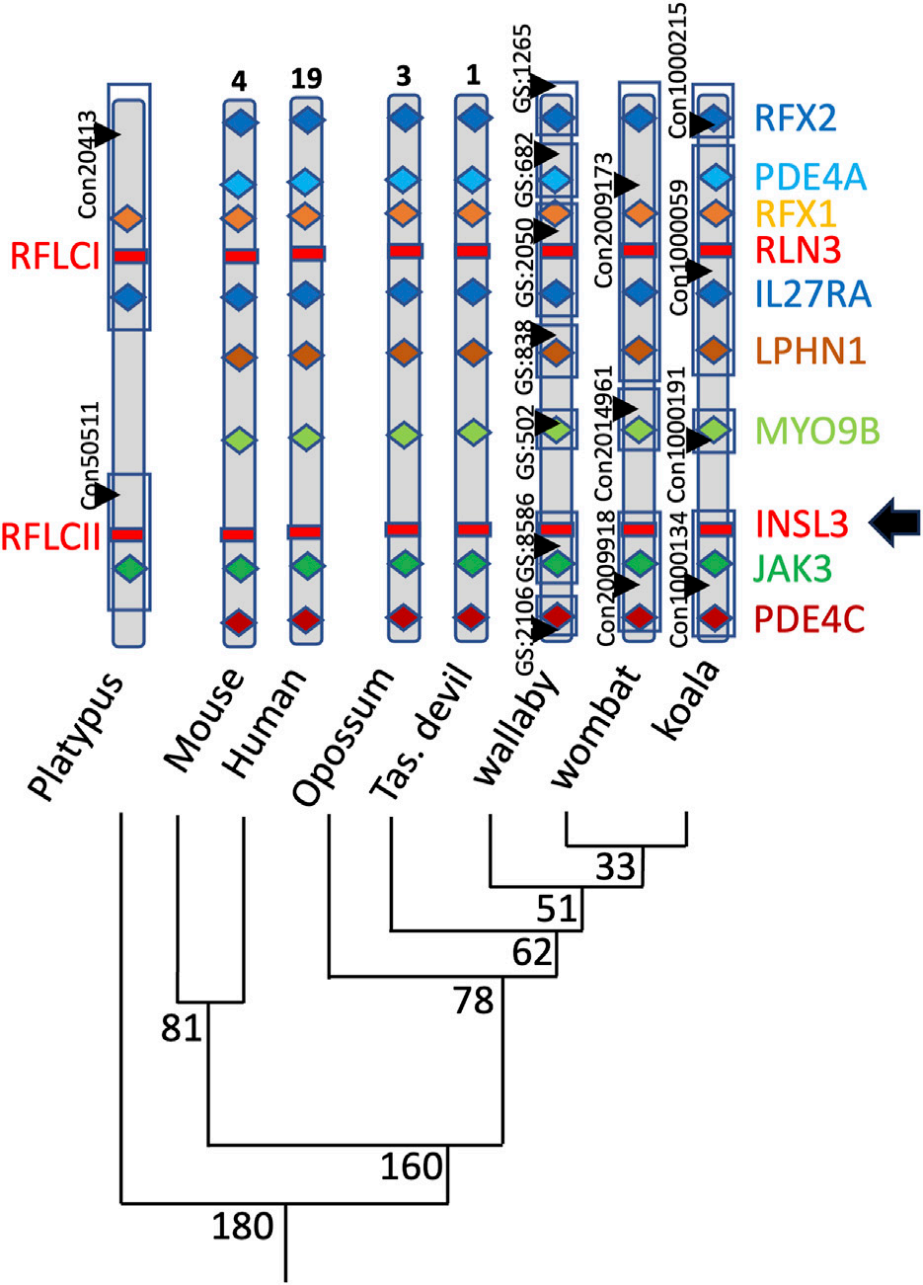
Position of INSL3 within the genomes of a range of marsupials. Mammalian *INSL3* genes are all located in the same position within the genome between the *Myosin 9B* and *Janus Kinase 3* genes, indicating a likely conserved regulation and function.

The genomic *INSL3* locus is also conserved in marsupials relative to eutherians and monotremes located between the Myosin 9B (MYO9B) and Janus Kinase 3 (JAK3) genes in koala, wombat, tammar wallaby, Tasmanian devil, and grey short-tailed opossum (Figure 4). The marsupial RXFP2 nucleotide and amino acid sequences were highly conserved with eutherian species and showed approximately 90% and 95% sequence identity among therians, respectively.

### INSL3 mRNA expression in the developing marsupial testes

**FIGURE 5:**
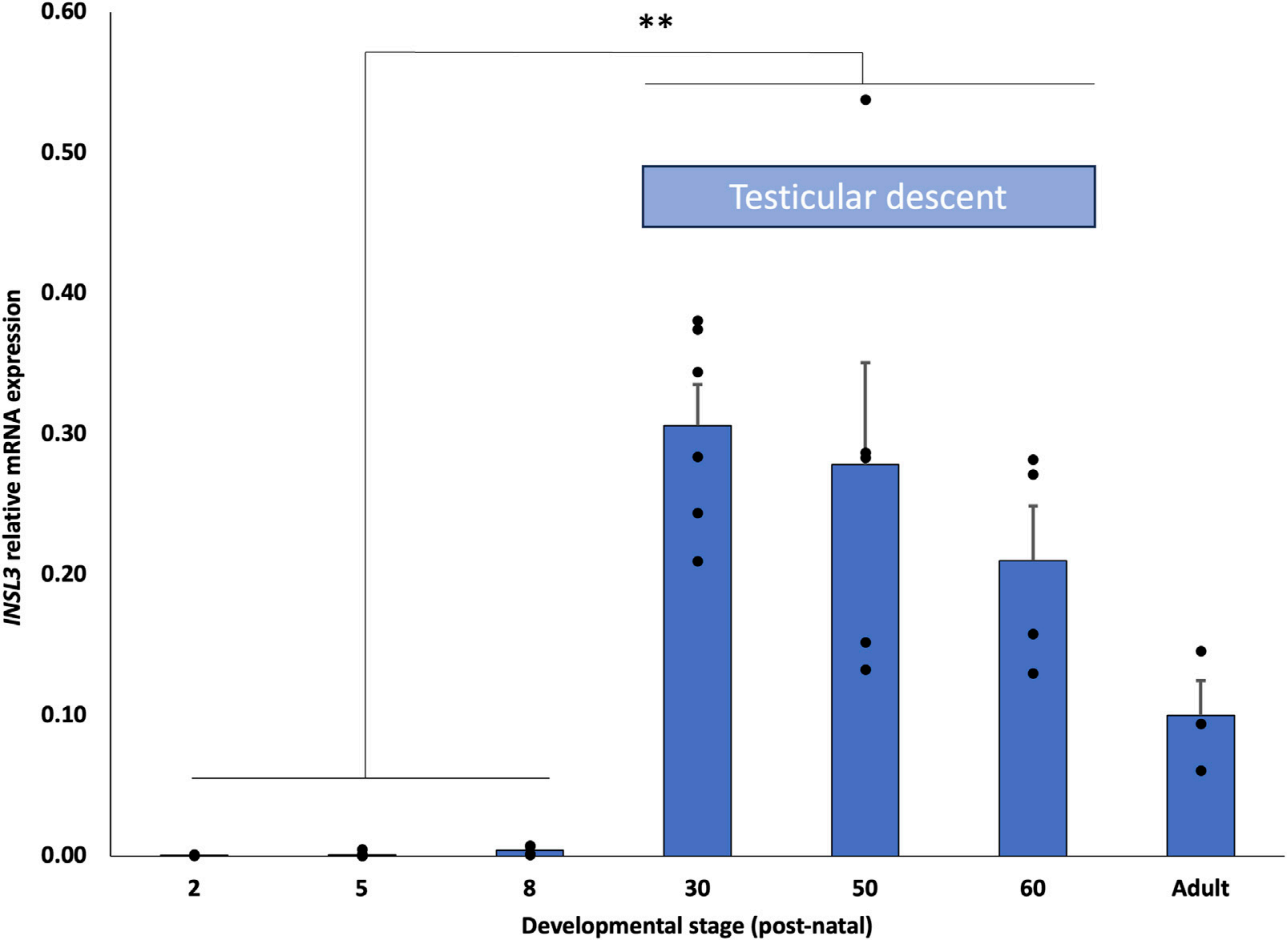
Developmental expression profile of *INSL3* mRNA in dunnart testis. *INSL3* mRNA expression is significantly elevated in developing marsupial testes during the critical period of testicular descent (D15–60 PP).

We compared *INSL3* mRNA expression at seven different developmental stages in the fat-tailed dunnart (*S. crassicaudata*), D2, 5, 8, 30, 50, and 60, and adults (D200+). Expression was barely detectable from D2–8 post-partum, a developmental stage prior to testis migration from the mesonephros (Figure 5). By D30, a developmental stage in the middle of transabdominal descent, the expression was significantly higher (*p* < 0.01) than in earlier stages and had the highest nominal expression throughout early development. The expression at D50 and D60, the later stages of testicular descent, was also significantly higher than at D2–8 but progressively lower and not statistically different from that at D30. The general relative expression pattern of *INSL3* was identical in two separate housekeeping genes, with the expression relative to TPB lower than that of PUM1 from D30 to adult stages (Supplementary Figure S1).

**FIGURE 6:**
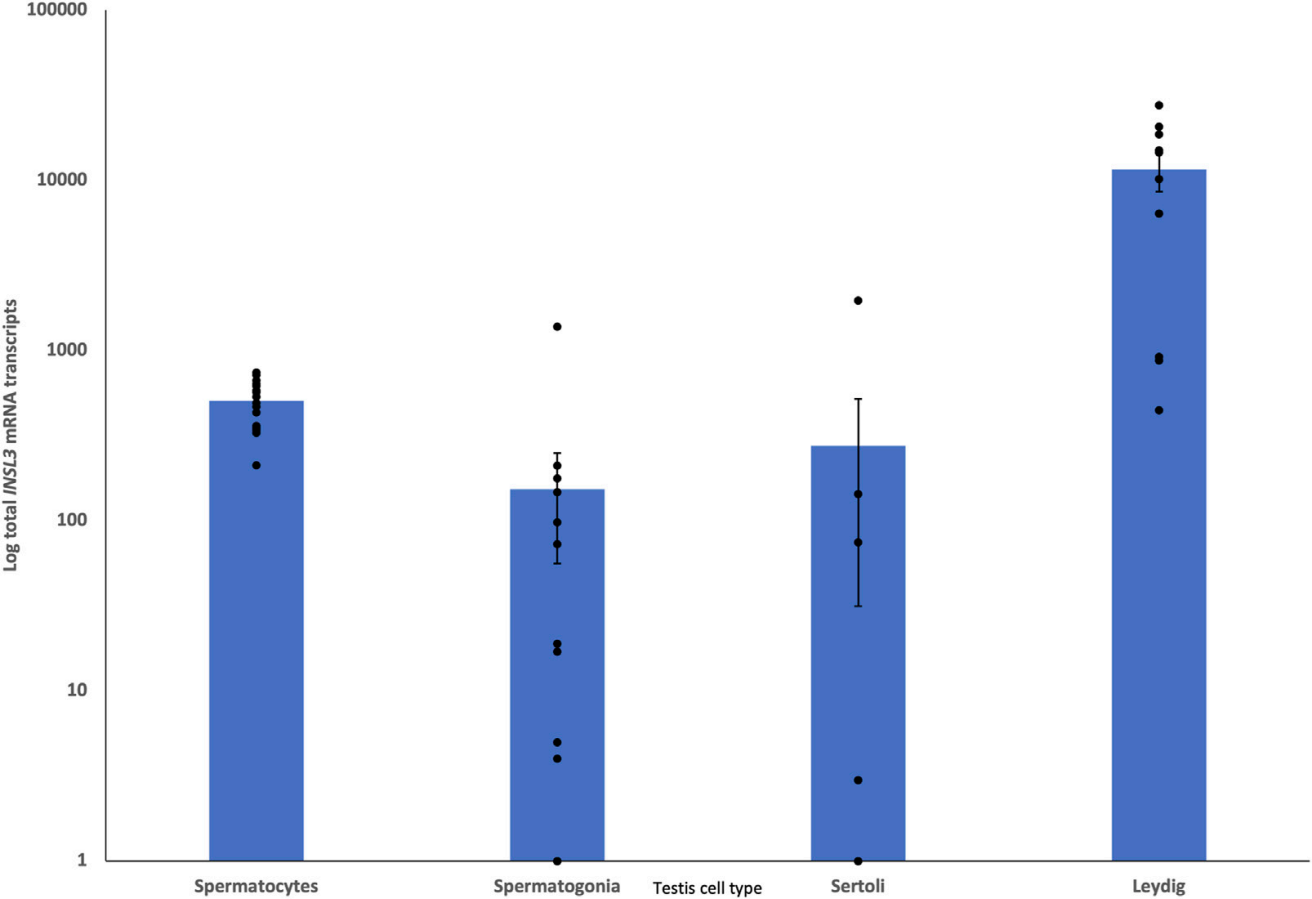
Single-cell RNA-seq comparison of presumptive testis cells for *INSL3* expression. *INSL3* expression is at least 30 times higher in Leydig cells than in other testis cell types, demonstrating that *INSL3* originates from a similar cell type (Leydig) as the eutherian species.

Single-cell RNA-seq data from juvenile and adult dunnart testes showed *INSL3* expression in presumptive Leydig cells that was ×30 greater than that in the next highest cell type (presumptive spermatocytes; Figure 6).

## Discussion

**FIGURE 7:**
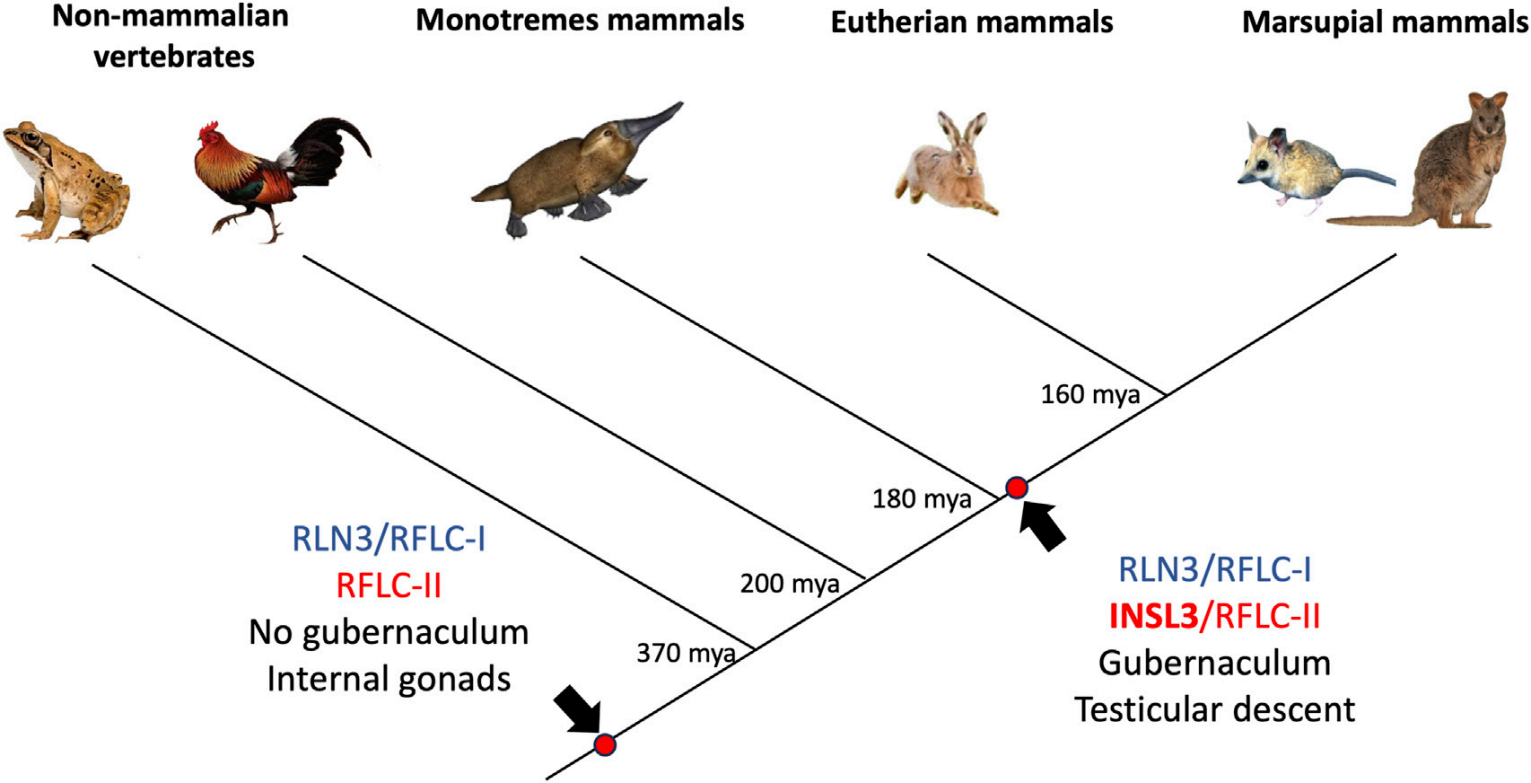
Phylogenetic reconstruction of the relaxin family locus (RFL) and the origin of INSL3/RXFP2-mediated testicular descent in vertebrates. Monotremes and non-mammalian vertebrates (after the split from tetrapods) possess two *RFLC* genes denoted as *RFLC-I* and *RFLC-II*. These genes encode *RLN3* and *INSL3* in therian mammals, respectively. However, the monotreme *RFLC-II* gene groups phylogenetically with the ancestral *RLN3* gene as opposed to all therian (eutherian and marsupial) *INSL3* genes, which suggests it arose independently in a therian ancestor to drive testicular descent (a character trait of 99.9% of therian mammals).

Taken together, these data provide considerable evidence for a therian origin of testicular descent driven by INSL3 and its receptor, RXFP2 (Figure 7). Genomic analyses show that a wide range of marsupial orders that have descended testes, namely, the Diprotodontia, Dasyuromorphia, Notoryctemorphia, Microbiotheria, and Didelphimorphia, have an *INSL3* gene located at the same genomic position as the Eutheria. Marsupial *INSL3* genes also group phylogenetically with eutherian *INSL3*, but not monotreme orthologs. The *INSL3* beta chain was very highly conserved among mammals (90% amino acid identity), probably because it is involved in RXFP2 receptor binding. Critically, all marsupials possess a key amino acid substitution in the beta chain, absent in monotremes, that confers specificity to RXFP2 as opposed to the ancestral INSL3 gene, which binds both RXFP-1 and -2 with low affinity (Park et al., 2008). The *RXFP2* gene was also highly conserved among all mammals (95% amino acid sequence identity), which may indicate a broader generalized function for RXFP2 in RLN/INSL signaling throughout the body.

In addition to the genomic evidence, we demonstrated the mRNA expression of *INSL3* in the developing testis and described the process of testicular migration and pouch/scrotum differentiation in this species. Importantly, the *INSL3* expression was predominantly from the Leydig cells and increased in the developing marsupial testes after D8 once they had started their transabdominal migration. This is consistent with data from the mouse and suggests that INSL3 is driving this process in an identical way in marsupials, by stimulation of gubernaculum growth (Zimmerman et al., 1999).

The gubernaculum connects the caudal epididymis of the testis to the scrotal wall and eventually pulls the testis into the scrotum due to its own growth and regression of the caudal suspensory ligament. The fact that marsupials appear to have the same molecular mechanism of transabdominal testicular descent and the presence of a scrotum, as in eutherians (i.e., growth of the gubernaculum via *INSL3/RXFP2* binding), but a different mechanism and location of scrotum differentiation (i.e., X-Ch dosage and pre-penial position), suggests that the presence of a scrotum may have originated in a therian ancestor, but then the position and mechanism of determination changed after the split between eutherians and marsupials. The alternative hypothesis is that the gubernaculum originally evolved to bring the testes to the abdominal wall, after which marsupials and eutherians developed analogous scrotal structures independently. Consistent with this hypothesis, the marsupial mole (*N. typhlops*), which has subdermal testes, presumably due to the evolutionary loss of the scrotum, still has a functional *INSL3* gene, which demonstrates the continued importance of this pathway in bringing the testes to this subdermal position.

In conclusion, our data provide strong evidence for a therian origin of testicular descent and confirm the evolutionary benefit of this mechanism given the retention of this trait in the majority of therian mammals, including all marsupials.

## Data Availability

The datasets presented in this study can be found in online repositories. The names of the repository/repositories and accession number(s) can be found in the article/Supplementary Material.
