# Supplementary figures and images for: Therian origin of INSL3/RXFP2-driven testicular descent in mammals
Source: Front Cell Dev Biol. 2024 Feb 2;12:1353598. doi: 10.3389/fcell.2024.1353598 (PMC10877632; doi:10.3389/fcell.2024.1353598)

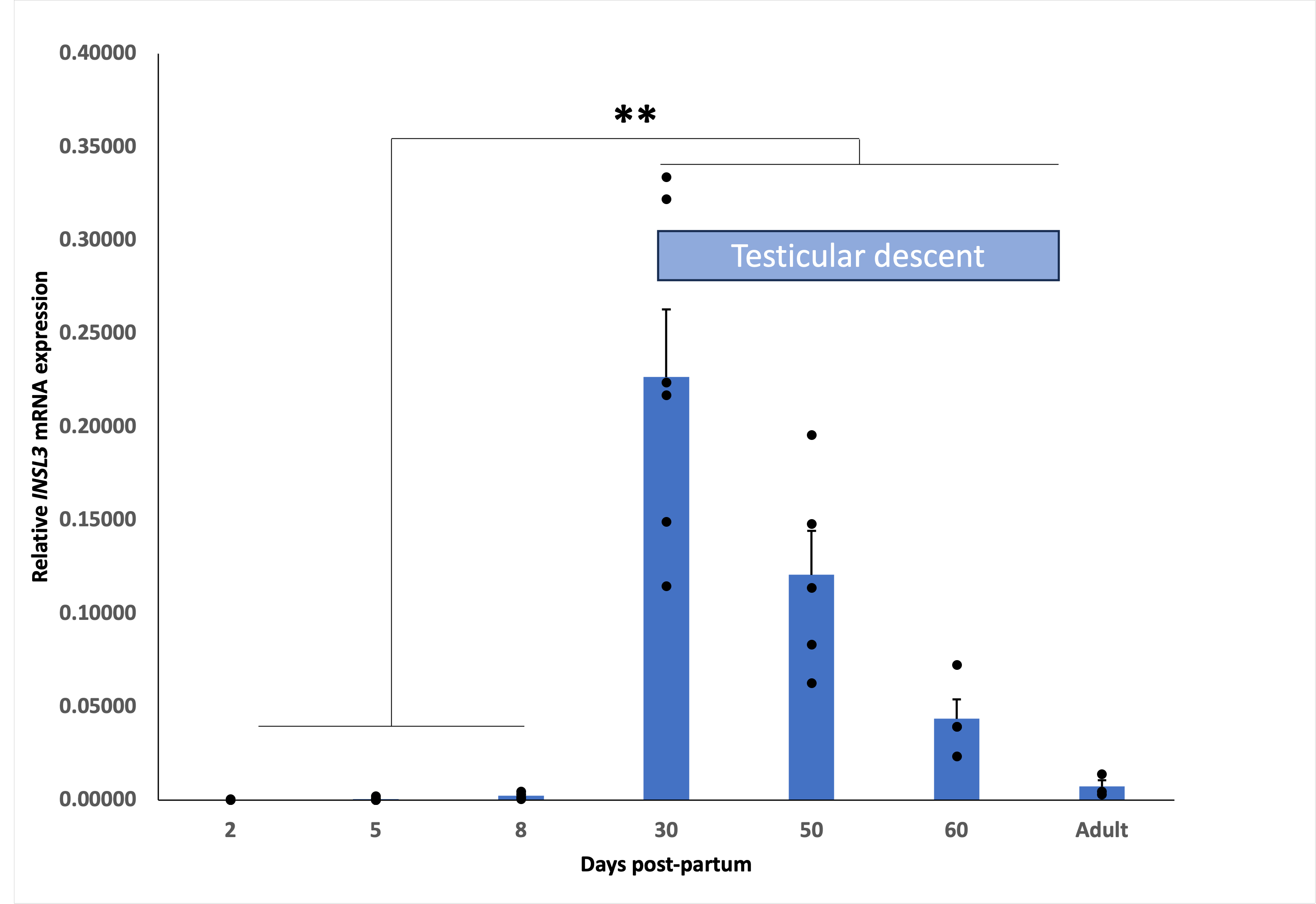

Supplement: Supplementary file 1 [file Image1.tiff]
